# A Compact High-Precision Cascade PID-Control Laser Driver for Airborne Coherent LiDAR Applications

**DOI:** 10.3390/s25092851

**Published:** 2025-04-30

**Authors:** Zixuan Ming, Xianzhuo Li, Yanyi Wang, Yuanzhe Qu, Zhiyong Lu, Honghui Jia, Haoming Yuan, Qianwu Zhang, Junjie Zhang, Yingxiong Song

**Affiliations:** 1 Key Laboratory of Specialty Optics and Optical Access Networks, Institute for Advanced Communication and Data Science, Shanghai University, Shanghai 200444, China; mingzixuan@shu.edu.cn (Z.M.); lxz_edu@shu.edu.cn (X.L.); quyuanzhe@shu.edu.cn (Y.Q.); zhangqianwu@shu.edu.cn (Q.Z.); zjj@staff.shu.edu.cn (J.Z.); herosf@shu.edu.cn (Y.S.); 2 Key Laboratory of Space Laser Transmission and Detection Technology, Shanghai Institute of Optics and Fine Mechanics, Chinese Academy of Sciences, Shanghai 201899, China; luzhiyong15@126.com (Z.L.); jiahonghui@siom.ac.cn (H.J.); 3 Teralink Optical Corporation, Shanghai 200443, China; yhm1283305307@163.com

**Keywords:** dual-frequency coherent doppler LiDAR, cascade PID control, frequency-temperature compensation mechanism

## Abstract

This paper solves the challenge of precise dual-frequency laser control in Airborne Coherent Doppler LiDAR systems by implementing an innovative laser driver architecture, which integrates compact hardware design with cascade Proportional-Integral-Derivative (PID) control and a frequency–temperature compensation mechanism. The experimental results demonstrate eminent performance with long-term temperature fluctuation below 0.007 °C, temperature stabilizing time under 4 s and long-term power fluctuation of the linear constant current source being <1%. The system enables wide-range temperature–frequency adjustment for individual lasers and dynamically adjusts the dual-laser beat frequencies between −1 GHz and +2 GHz, achieving the frequency difference fluctuation within 3 MHz. These achievements greatly enhance LiDAR performance and create possibilities for broader applications in dynamic environmental sensing, atmospheric monitoring, deep-space exploration, and autonomous systems.

## 1. Introduction

Since its booming in the 1970s, Light Detection and Ranging (LiDAR) technology has witnessed remarkable progression and diversifications in various fields [1,2,3,4]. The escalating sophistication of meteorological monitoring, autonomous navigation, topographic mapping, precision ranging, and spatial remote sensing [5,6,7,8,9] imposes demanding performance requirements on LiDAR systems. Airborne LiDAR, serving as a three-dimensional spatial information acquisition platform, facilitates rapid parameter extraction of terrestrial and atmospheric targets [10], with its distinctive operational paradigms and data processing methodologies garnering significant global research attention [11]. Based on Doppler principles, LiDAR measures target distances by analyzing time-of-flight (TOF) differences in reflected laser pulses [12], where the laser source serves as the critical component of the whole system. Laser performance fundamentally determines the resolution, stability, measurement accuracy, and real-time capabilities of LiDAR [13], with its output optical power regulated by driving current and its wavelength modulated by crystal temperature [14]. Hence, traditional laser drivers basically consist of constant current sources and thermal control circuits. Due to constrained resources and rigorous operational demands [15], airborne implementations confront heightened challenges. Contemporary LiDAR architectures are categorized into direct detection systems based on TOF principles [16,17] and coherent detection systems capable of simultaneous distance–velocity measurements with superior accuracy and interference immunity [18,19], the latter demonstrating preferential adoption in airborne applications despite elevated system complexity.

Recent advancements in laser driver design have demonstrated significant progress in temperature regulation and current stability. Li et al. (2018) introduced a novel low-noise, low-power laser driver with high modulation bandwidth integrating Hall–Libbrecht current control and ADN8834-based temperature stabilization [20]. The system demonstrated 523 ppm/°C temperature coefficient, ±0.0052 °C stability, and achieved <11.5 ppm CO_2_ and <0.124 ppm C_2_H_2_ detection limits via absorption spectroscopy. He et al. (2019) proposed a novel semiconductor laser power stabilization system utilizing a Microcontroller Unit (MCU) as the primary control unit, with LT3080 from ADI serving as the core component to establish a voltage-controlled constant current source [21]. The system integrates an operational amplifier and resistor–capacitor network to construct a power control loop, incorporating a neural network-based PI control algorithm to achieve short-term stable power output for semiconductor laser diode (LD) with <1 W output power and <1% long-term power stability. Zhao et al. (2022) developed a semiconductor laser current driver and temperature control system employing an STM32 microcontroller as the central processing unit [22]. The system features an operational amplifier and N-MOS transistor-based constant current source, implements a fuzzy PID-control algorithm and utilizes MAX1968 from ADI for thermoelectric cooler (TEC) driving. This configuration enables linear adjustment of output drive current from 0 to 100 mA with ±0.01 mA precision and ±0.005 °C temperature control accuracy. Gao et al. (2023) presented a semiconductor laser drive circuit consisting of an operational amplifier and power transistor for current regulation, coupled with MAX1978-based TEC control circuitry [23]. The system employs PID control to maintain a constant temperature, achieving an output wavelength standard deviation of 0.0002. Yu et al. designed a semiconductor laser driver circuit using field programmable gate arrays (FPGA) as control chips. The temperature control and feedback circuit use high-speed MOSFET as the switching device to drive the laser diode and TEC driving is achieved through ADN8834 and the fuzzy PID algorithm. The driver circuit achieves ±0.005 °C temperature control accuracy and ± 2 pm laser wavelength stability [24]. In general, most existing temperature control solutions rely on commercial off-the-shelf TEC driver ICs optimized for low-voltage applications, which become inadequate when driving high-voltage TECs integrated within airborne LiDAR systems. Furthermore, traditional approaches focusing solely on laser temperature regulation cannot inherently guarantee stable optical frequency output, necessitating complex calibration procedures, which greatly intensify system software complexity. Moreover, most current laser drivers prioritize singular performance factors such as temperature control accuracy, neglecting specific domain requirements of coherent LiDAR systems, resulting in a lack of operational adaptability and integration capability. Critically, the commonly used single-stage PID-control algorithm, which is effective for low-inertia, fast-response systems, exhibits inherent limitations in high-inertia, slow-response environments typical of airborne platforms, tending to result in control instability. To address these challenges, we propose a specialized control framework featuring a compact hardware design tailored for spatial constraints, a temperature cascade PID-control algorithm to accommodate high-inertia thermal environments, and dual-laser frequency difference regulation for simplified system architecture while maintaining precise frequency adjustment. These integrated implementations represent a pioneering systematic investigation into laser driver solutions specifically designed for dual-frequency coherent airborne LiDAR applications, overcoming hardware integration barriers and the accuracy limitations of temperature control using a comprehensive and cohesive design philosophy.

In this paper, we systematically elaborate on the design of a dual-channel seed laser driver specifically developed for airborne coherent LiDAR systems. Building upon the work of ACP2024 [25,26], through comprehensive research into the design of critical hardware components including precision constant current source circuits, TEC drive circuits, laser temperature monitoring systems and advanced control methodologies such as cascade PID-control algorithms, frequency sweeping techniques and frequency-temperature compensation strategies, we successfully designed and physically implemented the laser driver. Rigorous experimental evaluations demonstrate the exceptional performance characteristics of the driver: achieving temperature stabilization time < 4 s with long-term fluctuation <0.007 °C, corresponding to dual-laser frequency difference fluctuations maintained below 3 MHz; delivering a linearly controlled constant current source exhibiting long-term stability better than 1% deviation; enabling on-demand sinusoidal wavelength adjustment across user-defined spectral ranges; and facilitating flexible beat frequency adjustment between dual lasers within −1 and +2 GHz. Notably, integration of this driver into a coherent LiDAR system yielded successful static ranging measurements over a 2.73 km distance. The research outcomes not only provide critical hardware infrastructure for advanced airborne coherent LiDAR applications but also demonstrate significant potential for broader technological implementations.

The paper is organized as follows: Section 2 analyzes coherent LiDAR measurement principles and laser characteristics; Section 3 details hardware and software co-design of the laser driver; Section 4 presents experimental validation; Section 5 concludes with research contributions.

## 2. Principle

### 2.1. Airborne Coherent LiDAR Measurement Principle and Error Analysis

**Figure 1:**
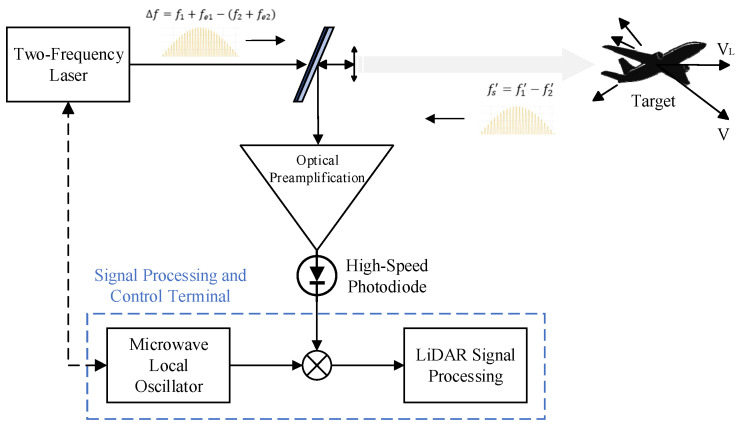
Optical carrier-based coherent LiDAR system.

Figure 1 depicts the dual-laser coherent Doppler LiDAR architecture implemented in this paper [27], integrating key modules including local reference laser (RL) and measurement laser (ML), dedicated drivers, optical coupler and data processing unit. The system employs independent seed lasers generating RL and ML beams with wavelength control managed by specialized drivers enabling 0-to-GHz-scale frequency difference tuning. Through the control of a balanced modulator and free-space optical link coordination, the signal processing unit dynamically adjusts the inter-laser frequency difference which is the critical parameter for subsequent data analysis. During operation, combined beams pass through a mixer before target illumination, with return signals collected by optics and converted to electrical signals via balanced detection. Laboratory validation utilizes acousto-optic frequency shifters and optical attenuators to emulate field detection conditions.

The purpose of employing dual lasers is to achieve a large detection range for the system. Assuming the actual output frequencies are $f_{1}$ and $f_{2}$, respectively, and considering the errors between the actual output frequencies and the desired frequencies of the individual lasers as $f_{e1}$ and $f_{e2}$, respectively, their frequency difference is given by:

(1) $$∆f=f_{1}+f_{e1}-(f_{2}+f_{e2})$$

Considering that the frequency difference falls within the microwave frequency range, allowing it to be effectively detected by the photodetector and enabling subsequent data processing. Considering the relative radial velocity $V_{r}$ between the LiDAR and the target, which is much smaller than the speed of light ($V_{r}\ll c$), the approximated Doppler shift expressions yield the echo frequencies $f_{1}^{\prime }$ and $f_{2}^{\prime }$ as follows:

(2) $$f_{1}^{\prime }=f_{1}+f_{e1}-2\frac{V_{r}(f_{1}+f_{e1})}{c}$$

(3) $$f_{2}^{\prime }=f_{2}+f_{e2}-2\frac{V_{r}(f_{2}+f_{e2})}{c}$$

The echo signal frequency $f_{s}^{\prime }$ obtained after the response of the balanced detector is:

(4) $$\begin{array}{l} \;f_{s}^{\prime }=f_{1}^{\prime }-f_{2}^{\prime }\\ \;\;\;=f_{1}+f_{e1}-(f_{2}+f_{e2})-2\frac{V_{r}(f_{1}+f_{e1})}{c}+2\frac{V_{r}(f_{2}+f_{e2})}{c}\\ \;\;\;=∆f(1-2\frac{V_{r}}{c}) \end{array}$$

From the above equation, it is evident that the echo signal frequency is solely dependent on the frequency difference between the two lasers and the relative radial velocity of the target. To enhance the detection accuracy and resolution of the LiDAR system, it is imperative to minimize the frequency errors of the lasers as much as possible. Although laser frequency fluctuations can be compensated to some extent by algorithms, it is still crucial to maintain high precision in the laser sources.

**Figure 2:**
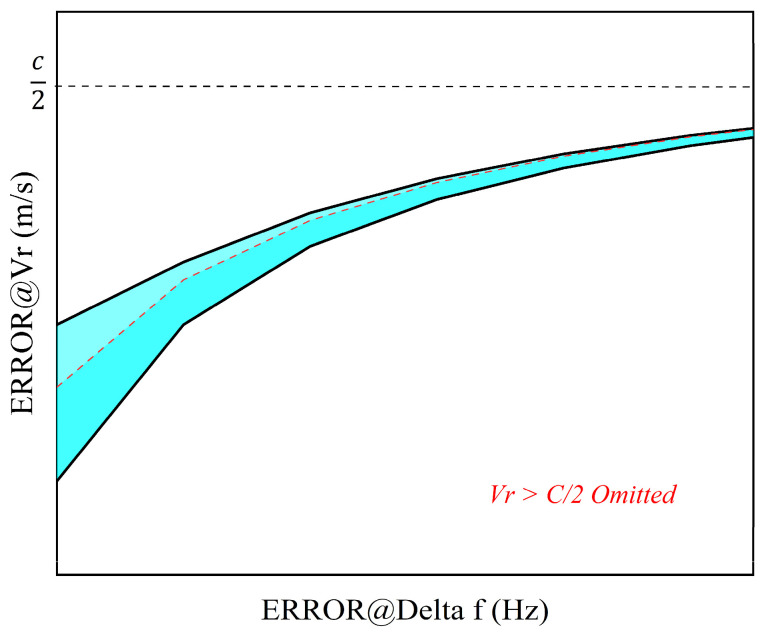
Relationship between frequency error and target velocity error.

Figure 2 illustrates the proportional relationship between the laser frequency error ($f_{e}$) and the velocity measurement error ($∆V_{r}$). With elevated target velocities ($V_{r}/c$ significant), Doppler shifts ($f_{s}\propto V_{r}/c$) dominate the frequency shift, reducing the relative contribution of laser frequency instability ($f_{e}/f$). This suppression of instability-induced errors strengthens with increasing velocity. In converse, diminished Doppler shifts cause laser frequency instability to dominate error propagation ($∆V_{r}\propto f_{e}\cdot c/f$) under low-velocity conditions ($V_{r}/c\approx 0$). The measurement inaccuracies are directly amplified by frequency instability, reflecting the transition from signal-dominated to noise-dominated regimes. These relationships collectively underscore the critical role of laser frequency stability in achieving precise velocity measurements across all operational scenarios of the entire LiDAR system.

To mitigate this velocity-dependent vulnerability and improve measurement robustness, the system employs a balanced photodetector with 150 MHz operational bandwidth, strategically selected to suppress high-frequency noise beyond the operational spectrum. When the Doppler frequency induced by target velocity exceeds the bandwidth of the photodetector, active adjustment of the dual-laser beat frequency through closed-loop control dynamically relocates the signal within the response window.

Besides the control stability, the tracking rate of the optical frequency also emerges as a key performance parameter that determines velocity tracking speed and system temporal resolution. The integrated high-power TEC achieves rapid adjustment of optical frequency. The TEC enables rapid intra-cavity thermal adjustment (dT/dt=2 °C/s max.), facilitating corresponding frequency tuning rates exceeding 500 MHz/s through the temperature–frequency coefficient (df/dt=10 GHz/s max). This thermal actuation capability establishes the TEC driver as a pivotal subsystem, particularly crucial for maintaining phase coherence in dynamic airborne measurement scenarios.

### 2.2. P–I–U and Temperature–Frequency Characteristics of the Laser

The laser utilized in the system is a single-frequency, all-solid-state laser with an output wavelength of 1064 nm [28]. It internally integrates modules and devices such as a TEC, laser crystal, piezoelectric ceramic (PZT), thermistor, and optical fiber. The laser emits light through a semiconductor laser (pump laser) with a central wavelength of 807.51 nm. The following mainly presents the P–I/U–I characteristics of the pump laser and the temperature–frequency tuning characteristics of the laser used in this system.

**Figure 3:**
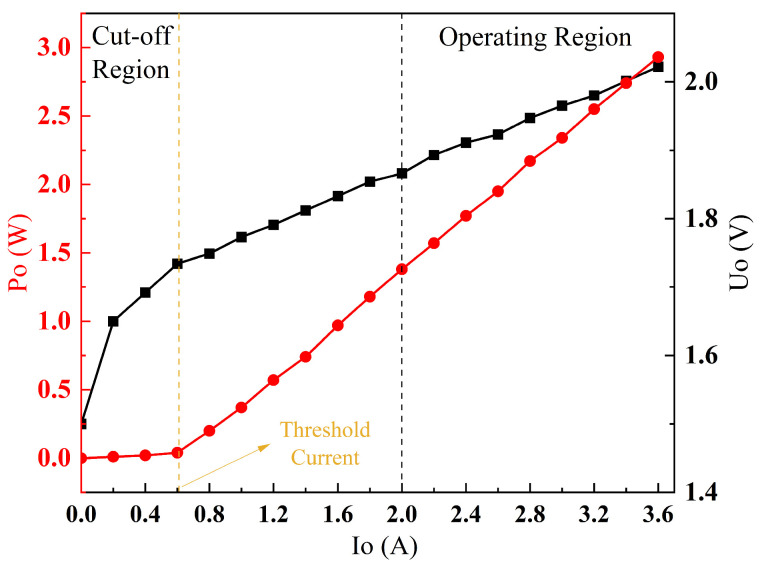
P–I/P–U curves of the pump laser.

Figure 3 showcases a comprehensive graph depicting both the P–I and U–I characteristics of the pump laser, illustrating the relationship between the output optical power and the injected current through the P–I curve, and the correspondence between the voltage across the pump laser and the injected current through the U–I curve. Below the luminescence threshold, the pump laser remains non-emissive; however, as the injected current exceeds this threshold, the pump laser gradually initiates the output laser with the P–I curve exhibiting an approximately linear relationship. Meanwhile, the voltage increases tardily at lower injected currents; as the injected current surpasses the threshold, the pump laser transitions to stable laser light output and the U–I curve begins to display a linear trend.

**Figure 4:**
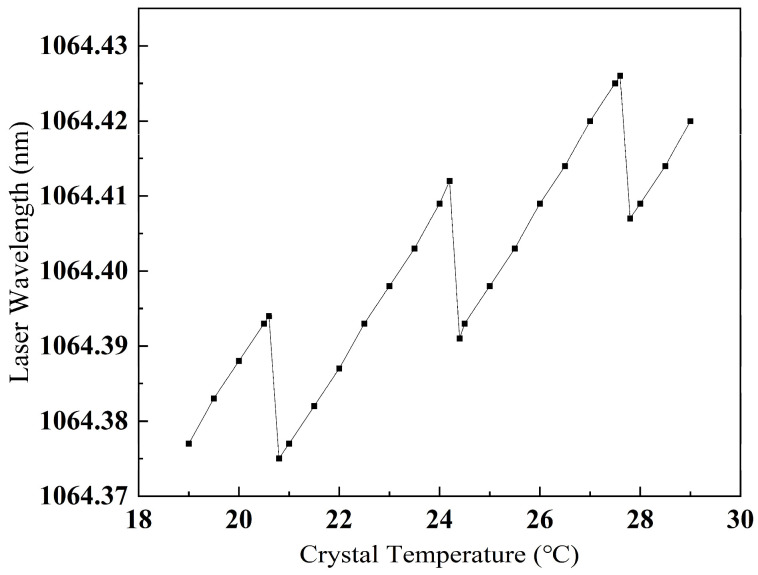
Variation of laser wavelength with temperature.

As previously discussed, the critical aspect of the laser lies in its frequency tuning performance which directly determines the temperature control range of the driver. Figure 4 presents the frequency curve of the output laser as the internal crystal temperature varies between 19 °C and 29 °C, revealing three distinct mode-hopping points at 20.6 °C, 24.2 °C and 27.6 °C. Based on the detection speed range requirement of −3 to 1 Mach for this coherent velocimeter LiDAR system, and considering a necessary margin for frequency tuning, the frequency difference range between the two lasers is established at −2 to 1 GHz. This necessitates a frequency tuning range of greater than 3 GHz for a single laser. By analyzing the temperature–frequency characteristic curve, the linear range of 24.4 °C to 27.6 °C is selected, with the center temperature for laser control set at 26 °C and the temperature control range defined as 24.5 °C to 27.5 °C. The corresponding theoretical frequency tuning range is approximately 8 GHz.

## 3. System Design

### 3.1. Overall Architecture

**Figure 5:**
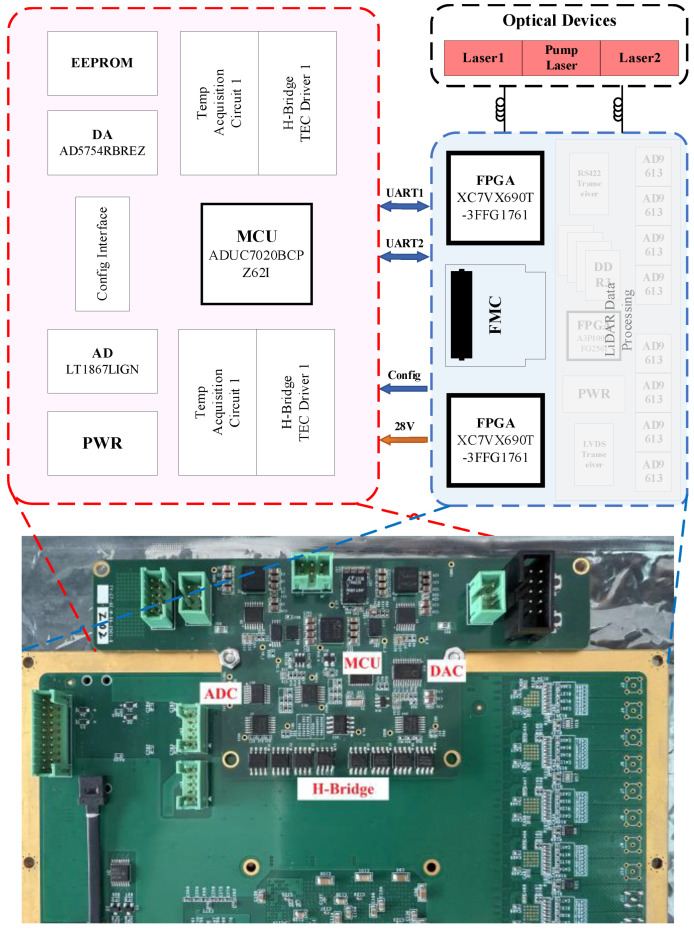
System overall architecture and completed assembly image.

The overall architecture of the laser driver is illustrated in Figure 5. In this configuration, the driver board functions as an autonomous unit, incorporating an independent microcontroller that serves as the main control chip, enabling it to operate independently. The FPGA-based data acquisition and processing board, meanwhile, is tasked not only with collecting and processing data from the LiDAR system but also with overseeing the control and the status of the driver board.

The system implements a cascade FPGA-MCU-based control architecture for Doppler-aided target tracking with integrated frequency regulation. Echo signal analysis via 65,536-point FFT peak detection quantifies the frequency offset between the received spectral peak and local oscillator reference, enabling real-time Doppler velocity estimation. This measurement dynamically adjusts the frequency-tracking setpoint of the PID controller, maintaining the echo signal centered within the 150 MHz detector bandwidth through cascade thermal PID modulation.

System power consumption totals approximately 70 W, dominated by the pump laser (42.3 W), high-speed frequency-sweeping ADC (18.7 W) and dual Xilinx Vertex UltraScale + FPGAs (8.604 W). To meet weak signal detection and real-time multi-channel processing demands, FPGA employs twelve parallel 65536-point FFT modules consuming 2520 18-Kb BRAMs and 252 DSP resources. The system maintains exceptional tracking accuracy while sustaining thermal equilibrium across the −40 °C to 85 °C operational range.

### 3.2. Hardware Implementations

**Figure 6:**
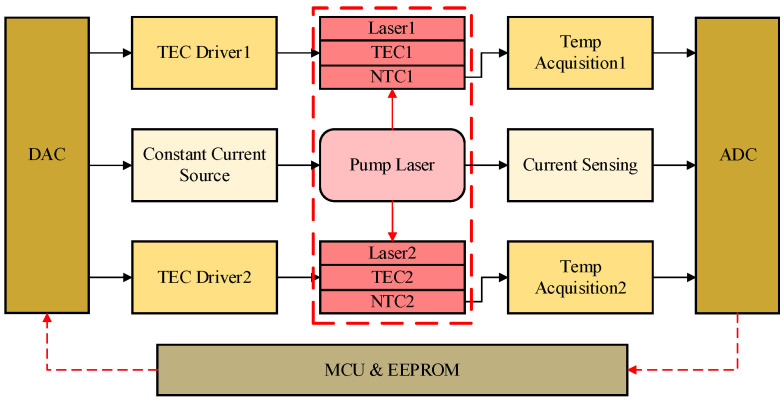
Laser driver hardware architecture.

The hardware system architecture of the laser driver is depicted in Figure 6. It comprises six primary components: a main control and storage module, Analog-to-Digital Converter (ADC) and Digital-to-Analog Converter (DAC) for parameter setting and data acquisition, two TEC drivers, two Negative Temperature Coefficient (NTC) resistance temperature sensing circuits, a constant current source, and its corresponding current sensing circuit. The power supply module is omitted from the figure for clarity.

#### 3.2.1. TEC Driver Circuit

**Figure 7:**
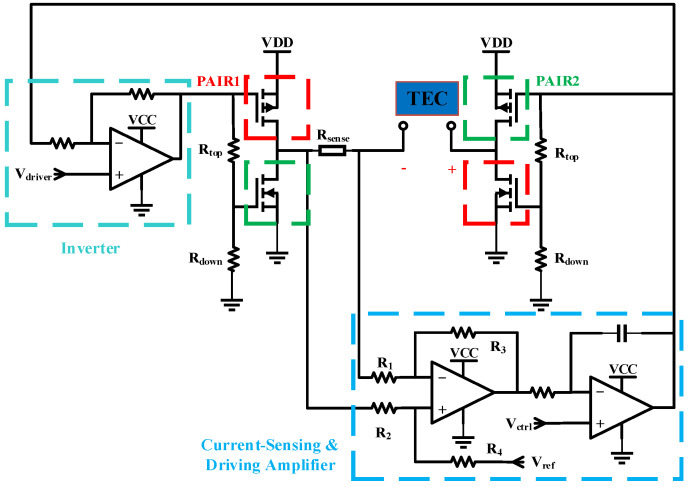
Detailed TEC driver circuit.

The driver circuit depicted in Figure 7 comprises an H-bridge configuration, integrated current sensing and driver circuitry and an inverter module. The H-bridge architecture is realized through the strategic combination of two N-channel MOSFETs and two P-channel MOSFETs, specifically selected for their rapid turn-on characteristics to minimize control latency. The source terminals of the P-MOSFETs are interfaced with the TEC power supply (VDD), which is meticulously generated via a high-efficiency switching power supply in conjunction with a Low Dropout Regulator (LDO). This dual-stage power conditioning ensures the delivery of a low-ripple, high-purity voltage supply, thereby enhancing the precision of temperature control operations. The drain terminals of the P-MOSFETs are interconnected with those of the N-MOSFETs, while their gate terminals are configured with voltage divider networks. The meticulous selection of MOSFET devices is critical to the performance of the driver circuit, ensuring strict symmetry in threshold voltages ($V_{th}$) between N-MOSFET and P-MOSFET pairs around the 0 V midpoint. This symmetry is vital for the H-bridge to achieve balanced thermal performance during both heating and cooling cycles. Through the precise assignment of the voltage dividing resistances ($R_{top}\;\&\;R_{down}$), the circuit enforces complementary switching logic. Ensuring that at any operational moment, one diagonal MOSFET pair within the H-bridge conducts while the opposing pair remains in a non-conducting state, thereby enabling seamless H-bridge commutation at all times.

The current-sensing and driver amplifier incorporates a sensing resistor, a high-precision differential amplifier and an integrator. During current conduction through the sensing resistor, the differential amplifier transduces the current into a voltage signal, producing an output symmetrically referenced to the midpoint voltage ($V_{ref}$). This processed signal is fed to the inverting input of the integrator. Concurrently, the non-inverting input of the integrator receives a control voltage from the DAC, which exhibits linear correspondence with the target current range for the TEC. The integrator functions dualistically as both a driver stage and a low-pass filter, effectively attenuating abrupt voltage transients. Its output voltage, ranging from 0 V to the supply voltage (VCC) of the operational amplifier, governs the switching behavior of the MOSFETs in the right half-bridge. An inverter module provides complementary control signals to the left half-bridge MOSFETs, ensuring mirrored switching states across the H-bridge.

The component values and voltage settings within the driver circuit are accurately calibrated based on the specific parameters of the integrated TEC. The discrete and modular design of the TEC drive circuit enables a scalable power supply, making it suitable for driving high-power TECs. The TEC electrical parameters integrated within the laser driver system are enumerated in Table 1.

Given $V_{max}=9.6\;V$, the TEC driver supply voltage (VDD) is set to 8 V. N-MOSFETs and P-MOSFETs with typical threshold voltages of $V_{th}=\pm 2.5\;V$ are selected, and the voltage divider resistors ($R_{top}$, $R_{down}$) are both set to 100 KΩ. The parameters of the TEC driver are configured as detailed in Table 2.

The non-inverting input of the inverter is set to 4.5 V, half of the supply voltage (VCC = 9 V) of the operational amplifier, ensuring symmetry between the inverting input voltage and output voltage of the inverter. The sensing resistor ($R_{sense}$) and the subsequent comparator output ($V_{diff}$) satisfy the following relationship:

(5) $$V_{diff}=R_{sense}\cdot I_{TEC}\cdot G_{comp}+V_{ref}$$

where $G_{comp}$ is the gain of the differential amplifier, $I_{TEC}$ is the current flowing through the TEC, and $V_{ref}$ is the reference voltage. For design simplicity, the sensing resistor and comparator gain are set to satisfy:

(6) $$R_{sense}\cdot G_{diff}=1$$

With $R_{sense}$ set to 50 mΩ, $R_{1}$ to $R_{4}$ adhere to the proportional relationship:

(7) $$R_{3}=R_{4}=20R_{1}=20R_{2}$$

Substituting these constraints, Equation (7) can be simplified to:

(8) $$V_{diff}=I_{TEC}+V_{ref}$$

Thus, the TEC control current and output control voltage relate as follows:

(9) $$I_{TEC}=V_{diff}-V_{ref}$$

**Figure 8:**
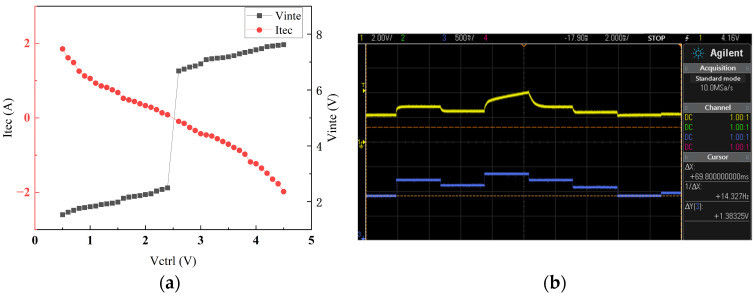
(**a**) TEC voltage–current relationship; (**b**) control voltage and integrator output voltage waveforms.

By setting the reference voltage $V_{ref}$ to the midpoint of the DAC output range, a linear correspondence between the control voltage and the current powering TEC is achieved. Ideally, the control voltage, integrator output voltage, inverter output voltage, and TEC current exhibit a linear relationship. However, practical curves may deviate due to parameters such as MOSFET threshold voltages and operational amplifier offset voltages. The measured voltage–current curve is shown in Figure 8a. Figure 8b demonstrates that the integrator output voltage changes smoothly with control voltage adjustments, ensuring proper H-bridge operation.

#### 3.2.2. Temperature Acquisition Circuit

**Figure 9:**
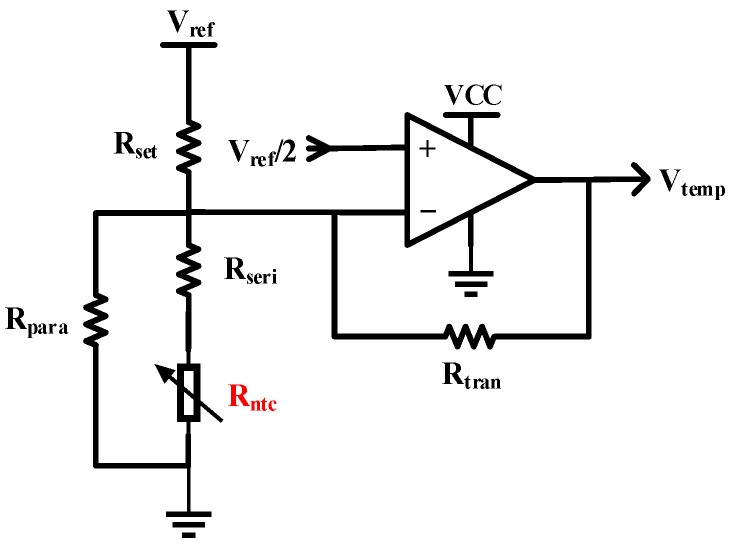
Detailed temperature acquisition circuit.

As depicted in Figure 9, the temperature sensing circuit comprises a rail-to-rail operational amplifier, a series linearizing resistor ($R_{seri}$), a parallel linearizing resistor ($R_{para}$), an NTC thermistor ($R_{ntc}$), a center temperature setting resistor ($R_{set}$), and a transimpedance resistor ($R_{tran}$). All resistors in this section are selected as low-temperature-coefficient precision resistors, and the operational amplifier is a precision rail-to-rail type to ensure measurement accuracy. The internally integrated NTC thermistor in the laser is a 10 K ± 1% thermistor with a B-value of 3950, exhibiting an exponential temperature–resistance characteristic curve. To linearize the temperature–resistance relationship within the range of 24.5–27.5 °C, centered symmetrically around 26 °C, appropriate series and parallel linearizing resistors are configured. The center temperature setting resistor $R_{set}$ should satisfy the following relationship:

(10) $$R_{set}=\left(R_{ntc@26\;{}^{\circ}C}+R_{seri}\right)//R_{para}$$

Magnitude of the transimpedance resistor $R_{tran}$ determines the temperature acquisition range:

(11) $$R_{tran}=\frac{R_{set}\cdot R_{N}}{R_{set}-R_{N}}$$

(12) $$R_{N}=\left(R_{ntc@27.5\;{}^{\circ}C}+R_{seri}\right)//R_{para}$$

The output voltage $V_{temp}$ of the temperature acquisition circuit can be calculated as:

(13) $$V_{temp}=V_{ref}\left(1+\frac{R_{tran}}{R_{N}}-\frac{R_{tran}}{R_{set}}\right)$$

In this paper, the center setpoint corresponds to a nominal thermistor resistance ($R_{ntc@26\;{}^{\circ}C}$) of 9567 Ω and the upper temperature limit corresponds to 8597 Ω ($R_{ntc@27.5\;{}^{\circ}C}$). To ensure effective linearization, the series ($R_{seri}$) and parallel ($R_{para}$) linearizing resistors are set to 2 kΩ and 1 MΩ, respectively. The resistor network configuration yields $R_{set}=11,434\;\Omega$. Following analogous computational procedures, $R_{tran}$ is determined to be 207,922 Ω. The actual measuring range is verified to be 25.3 °C to 27.5 °C with negligible nonlinearity errors, demonstrating compliance with the design specifications.

From the perspective of the output voltage, despite the adopted measures, the resistance is not strictly symmetrical around the center temperature. Additionally, temperature calibration is typically required when using NTC thermistors for accurate temperature measurement [29]. However, in the practical application of this study, only the constant frequency difference between the two lasers, i.e., the constant temperature difference, needs to be maintained. Therefore, it is unnecessary to consider whether the temperature and frequency of a single laser strictly matches. The temperature data collected here are merely relative values. This dual-laser frequency–temperature compensation mechanism significantly alleviates hardware design constraints and software computational burdens.

#### 3.2.3. Constant Current Source Circuit

With reference to the P–I–U characteristics of the pump laser shown in Figure 3, the parameters of the constant current source are configured as detailed in Table 3.

**Figure 10:**
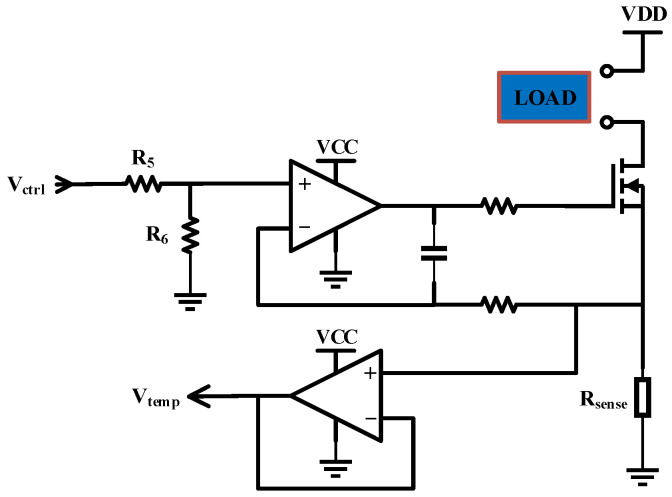
Detailed constant current source circuit.

As shown in Figure 10, the constant current source circuit employs an NMOS transistor, two operational amplifiers, and other resistive and capacitive components. This circuit is a negative feedback circuit, where the load current is continuously fed back through the sampling resistor $R_{sense}$. When the load current increases, the voltage at the inverting input of the operational amplifier becomes higher than that at the non-inverting input, causing the operational amplifier to output a low level, which turns off the NMOS transistor, thus reducing the load current. Conversely, when the load current decreases, the voltage at the inverting input drops below that at the non-inverting input, causing the operational amplifier to output a high level, which turns on the NMOS transistor, thereby increasing the load current. When a stable state is reached, the voltages at the inverting and non-inverting inputs of the operational amplifier are equal, and the gate voltage of the NMOS transistor remains constant, resulting in a stable current.

The resistance of the sampling resistor $R_{sense}$ is typically set to the milliohm level, resulting in an extremely small inverting input voltage, not exceeding 100 mV. Therefore, the main purpose of R5 and R6 is to attenuate the control voltage, enabling full utilization of the DAC output dynamic range. The sampling voltage, after passing through an Op-Amp Follower, is acquired by an ADC. The relationship between the control voltage and the load current is given by:

(14) $$I_{load}=\frac{R_{sense}*R_{6}}{R_{5}+R_{6}}*V_{ctrl}$$

### 3.3. Control Logic Implementations

#### 3.3.1. Cascade PID Control

**Figure 11:**
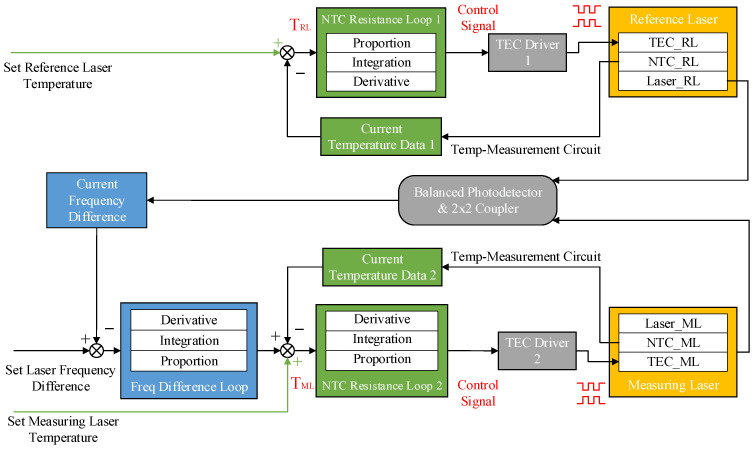
Single-stage and cascade PID structure.

The primary control logic employed in this study utilizes cascade PID control, with its structure illustrated in Figure 11. The cascade frequency difference control algorithm is primarily used for the control of the measuring laser, whereas the reference laser maintains a certain temperature after initialization, relying solely on single-stage PID for temperature control. In its specific implementation, two traditional PID controllers are used for cascade control, with the frequency difference PID controller serving as the outer loop control, i.e., the primary circuit, and the temperature PID controller serving as the inner loop control, i.e., the secondary circuit. The output value of the outer loop serves as the input for the inner loop.

The single-stage PID still employs the traditional discrete anti-integral windup PID algorithm to achieve precise temperature regulation. The PID controller generates a control signal by calculating the linear weighted sum of proportional, integral and derivative errors, thereby adjusting the current of the TEC driver to keep the temperature closely near the setpoint. The output control voltage is:

(15) $$V_{out}\left(k\right)=K_{p}e\left(k\right)+aK_{i}e_{i}\left(k\right)+K_{d}\left[e\left(k\right)-e\left(k-1\right)\right]$$

(16) $$e_{i}\left(k\right)=K_{i1}e_{i}\left(k-1\right)+K_{i2}e\left(k\right)$$

(17) $$a=\left\{\begin{array}{c} 1\;\left|e\left(k\right)\right|\leq \varepsilon \\ \;0\;\left|e\left(k\right)\right|>\varepsilon \; \end{array}\right.$$

where $e\left(k\right)$ represents the error between the current temperature and the set temperature, and a represents the threshold for the integral error. The tuning of the PID parameters is closely related to the heating and cooling efficiency of the controlled TEC, significantly impacting the time required for temperature stabilization and the overall stability of temperature control. Therefore, parameter tuning is necessary based on actual conditions.

**Figure 12:**
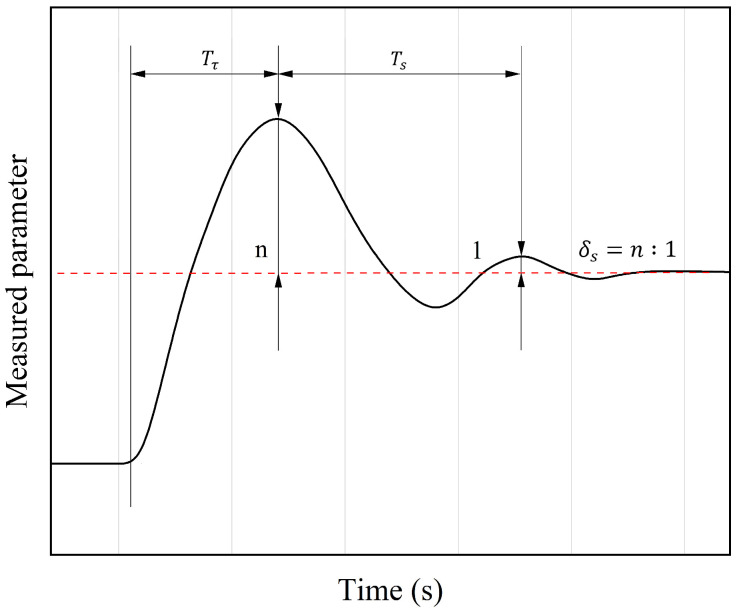
Transient attenuation curve under a setpoint step change.

There are various methods for PID parameter tuning including the empirical formula method, Ziegler–Nichols step response method, Cohen–Coon method, as well as numerous intelligent adaptive tuning methods integrated with other algorithms [30]. In this paper, the empirical formula method is adopted for cascade PID tuning to obtain optimal PID parameters rapidly and conveniently with reference to Figure 12 as follows:

- Set the integral time of both the primary controller (outer frequency loop) and secondary controller (inner resistance loop) to maximum and set the derivative time to zero; then, operate the cascade control system;
- Set the proportional band of the primary control to 100% scale and tune the secondary loop according to a specific damping ratio (commonly $\delta_{s}$ = 4:1 or 10:1). During tuning, gradually decrease the proportional of the secondary controller from a large value to determine the damping proportional band ($\delta_{2s}$) and damping oscillation period ($T_{2s}$) of the secondary controller;
- Fix $\delta_{2s}$, tune the primary loop by the same token to determine $\delta_{1s}$ and $T_{1s}$;
- Select the appropriate empirical formula based on the obtained values according to the chosen $\delta_{s}$ to calculate the PID parameters;
- Re-observe the cascade PID response to further fine-tune the parameters if necessary.

**Figure 13:**
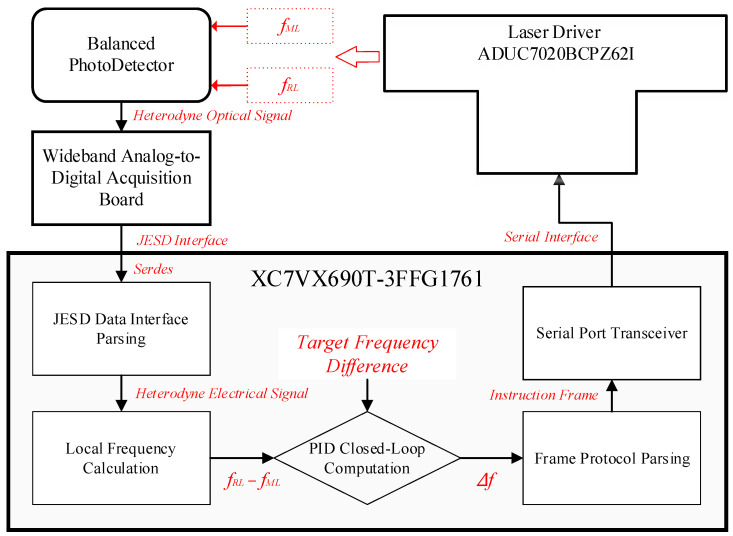
FPGA processing routing of frequency difference signals.

As shown in Figure 13, the TEC-controlled LD adjusts its output frequency by varying the internal crystal temperature. The optical frequency of the reference laser ($f_{RL}$) and the measuring laser ($f_{ML}$) are coherently mixed in an optical coupler, generating a heterodyne beat signal detected by a balanced photodetector optimized for high SNR due to dominant local oscillator power. The analog signal is digitized by an FMC Wideband RF Transceiver board and transmitted to an FPGA via JESD-protocol interfaces. Post-decoding, the system extracts the mixing center frequency ($f_{cen}$) and down-converted I/Q time-domain signals. Applying FFT to the I/Q data yields a signed frequency offset ($f_{het}$), enabling reconstruction of the local frequency as $f_{loc}=f_{cen}+f_{het}$ for ultra-wide-range signal monitoring. The FPGA-embedded PID controller computes the real-time desired frequency offset setpoint, which is frame-formatted and communicated to the laser driver, achieving closed-loop frequency offset control. This integrated system demonstrates precise frequency stabilization and dynamic range enhancement through coherent optical mixing and digital signal processing.

#### 3.3.2. Control Link Implementation

The laser driver system employs a high-precision analog microprocessor from ADI as the primary controller for analog temperature PID computations. As the core of the coherent LiDAR system, the data acquisition and processing board is equipped with XC7VX690T-3FFG1761 FPGA from Xilinx, which manages high-speed ADC data streams for real-time processing while concurrently executing analog frequency difference PID calculations for laser temperature stabilization. To enhance control resolution, the system integrates 16-bit ADCs/DACs with ±5 V input/output ranges.

**Figure 14:**
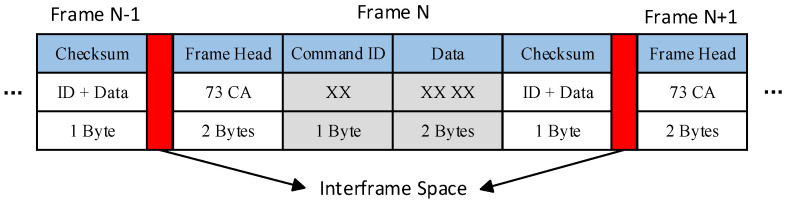
Instruction and data frame format.

The microcontroller and FPGA communicate via dual serial ports configured in primary backup redundancy. As illustrated in Figure 14, the instruction and data frame format for bidirectional communication is structured as follows:

- Frame Header: Marks the start of data transmission;
- ID Bit: Identifies the controlled parameter (TEC1/TEC2 temperatures, pump current, TEC current limit, control cycle, or other PID parameters);
- 2-Byte Data Field: Encapsulates specific control values;
- Checksum: Byte-wise sum of ID and data bits for transmit error detection.

The response frame mirrors the instruction format, with the ID bit indicating the controlled object (TEC1/TEC2/current source) and the data field validating instruction execution. Frames are transmitted at adjustable intervals (1 ms minimum) to ensure efficient data throughput, with the cyclic acquisition routine of the microcontroller enforcing a minimum 3 ms control period. All operational parameters remain persistently configurable via host terminal commands, and the onboard EEPROM of the laser driver ensuring parameter retention across power cycles. This hybrid analog–digital control architecture achieves precise frequency stabilization while maintaining system flexibility and reliability for advanced coherent detection applications.

## 4. Experimental Setup and Results

**Figure 15:**
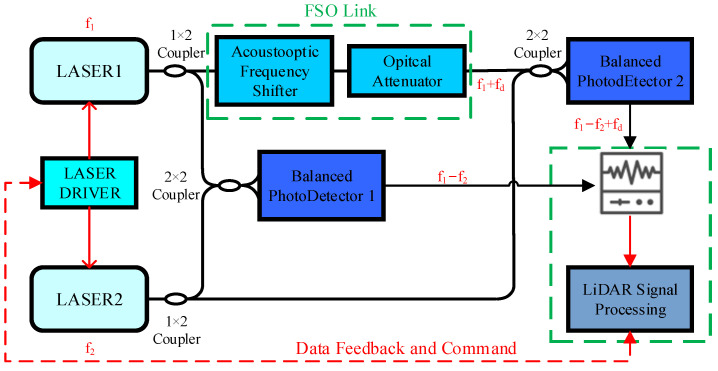
Experimental diagram of the dual-frequency coherent laser radar system.

The laboratory-simulated dual-frequency coherent LiDAR system, as shown in Figure 15, integrates a data acquisition and processing board that serves as the primary terminal for controlling the laser driver. The FPGA, through a serial port, sends commands to flexibly regulate the operational state of the driver board. An oscilloscope provides real-time visualization of the current laser frequency values and their minute fluctuations, offering a direct basis for assessing system performance. The pump laser simultaneously outputs pump light to two solid-state lasers. The laser driver used in this study features two independent temperature control loops to manage the frequency tuning of the lasers.

### 4.1. PID Response

**Figure 16:**
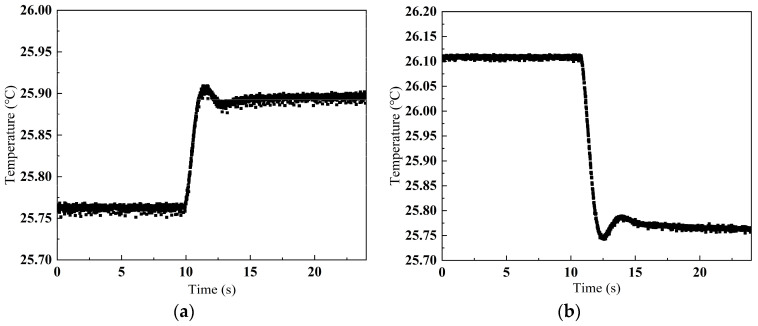
Temperature variation curve over time with single-stage PID controller: (**a**) 25.76 °C to 25.89 °C; (**b**) 26.11 °C to 25.76 °C.

Under ambient conditions of 15°C, temperature control commands were transmitted directly from the terminal to modulate laser temperature. The laser driver transmits real-time temperature data to the control terminal through serial interface, with its intervals set to 1 ms. Following preliminary data processing by FPGA, the data are transmitted via a serial port to debugging software which enables visualization and data storage. Figure 16 illustrates the dynamic response:

1. Cooling Phase: Initial setpoint = 25.76 °C. Post-command, the driver induced a controlled temperature decrease, stabilizing at 25.89 °C. Assuming linear temperature-data correlation, this ΔT ≈ 0.2 °C corresponded to a laser frequency tuning of ~400 MHz.
2. Heating Phase: Subsequent setpoint = 26.11 °C. The driver then drove a temperature increase, stabilizing at 25.76 °C. With ΔT ≈ 0.4 °C, the frequency shift reached ~1 GHz.

Critical performance index:

- Stabilization Time: <4 s for both heating and cooling transitions
- System Response: Minimal overshoot (<0.03 °C) and low steady-state error

**Figure 17:**
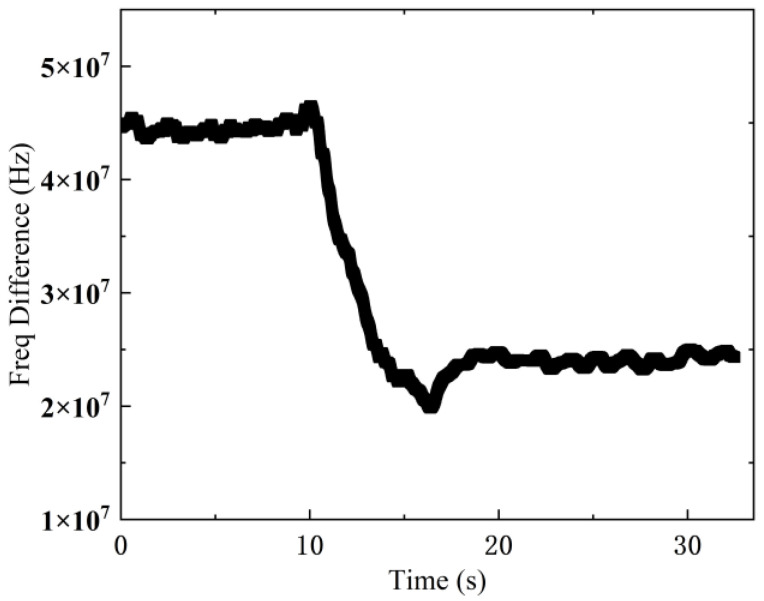
Temperature variation curve over time with cascade PID controller.

The cascade PID controller is adopted to maintain the optical frequency difference between RL and ML within the effective detection bandwidth of the balanced photodetector. Under ambient conditions of 15 °C and fixed reference laser frequency, the FPGA control terminal actively dispatched frequency adjustment commands to the laser driver board and the parameter of ML as illustrated in Figure 17.

Dynamic Response Characteristics:

1. Initial Stabilization:
  - Starting frequency offset: ~45 MHz
  - Post-control stabilization: ~24 MHz
  - Observed behavior: Progressive frequency reduction with minimal transient oscillations.
2. Post-PID Optimization (FPGA Parameters Tuned):
  - Frequency stabilization time: <5 s
  - System response: Slight overshoot and residual error.

Critical Performance Index:

- Stabilization Time: <5 s (post-PID parameter adjustment)
- Control Accuracy: <1 MHz steady-state error
- System Efficiency: >80% frequency offset reduction from initial state.

### 4.2. Voltage-Controlled Constant Current Source

**Figure 18:**
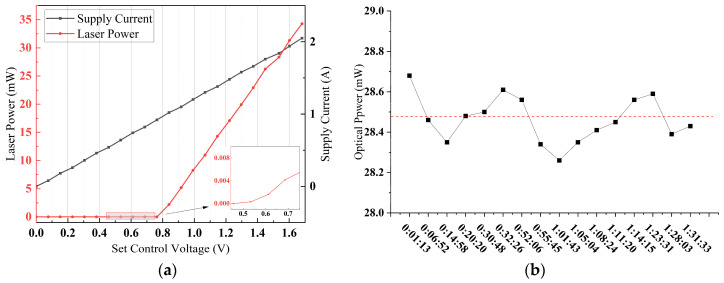
(**a**) Relationship between set control voltage, supply current and laser power; (**b**) optical power fluctuation over 90 min.

Under ambient conditions of 15 °C, the pump laser current was modulated via high-precision voltage control of a constant current source. The resultant laser power scaling with current injection is illustrated in Figure 18a, revealing the following:

1. Threshold Behavior: A clear lasing threshold at $I_{th}=0.61\;A$.
2. Linear Response: Above threshold, optical power exhibited a linear relationship with injection current (P∝I), in accordance with the theoretical characteristics presented in Figure 3.

Subsequently, the long-term stability was evaluated at $I_{supply}=1.8\;A$. The stability of optical power is quantitatively demonstrated by the root-mean-square error (RMSE) and instability param (γ) as follows:

(18) $$RMSE=\sqrt{\frac{1}{n}\sum_{i=1}^{n}{\left(y_{i}-{\hat{y}}_{i}\right)}^{2}}$$

(19) $$\gamma =\frac{y_{max}-y_{min}}{\bar{y}}\cdot 100\%$$

Following systematic calculations, the RMSE of optical power fluctuations is determined to be 0.1435 mW, corresponding to the long-term power instability below 1%.

### 4.3. Target Scanning and Tracking

The system functionality is divided into two primary stages: target scanning and target tracking. This experimental configuration (temperature sweeping) specifically simulates the behavior of the driver during the scanning phase. Under ambient conditions of 15 °C, the control terminal issues commands to maintain constant RL frequency while periodically modulating the temperature of the measurement laser. Concurrently, the data acquisition and processing terminal performs intermittent calculations at predefined frequency offset points to estimate the target relative velocity.

**Figure 19:**
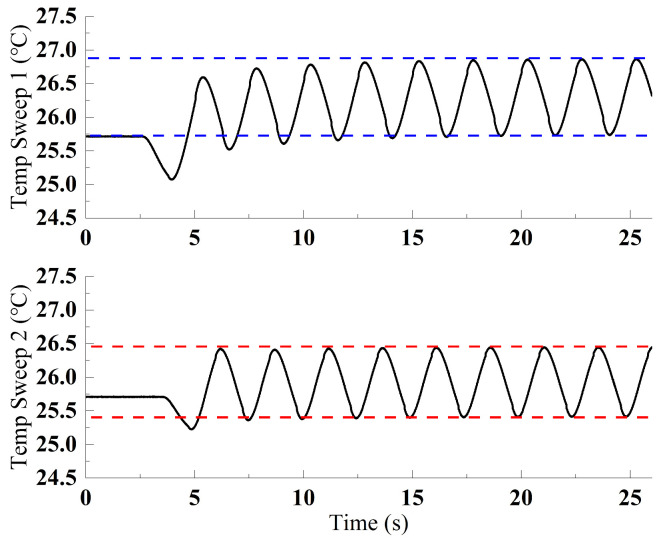
Temperature sweeping curves with different amplitudes.

Through systematic adjustment of command temperature parameters, inter-command intervals, and the heating/cooling coefficients of the TEC driver, the period and amplitude of the frequency sweeping curve can be dynamically optimized. Figure 19 demonstrates the system response: Upon command initiation, the laser transitions from a stable state to a frequency-sweeping regime within 5 s, achieving frequency tuning ranges of 3.2 GHz and 2.3 GHz during heating and cooling cycles, respectively.

Upon successful echo signal detection by the FPGA control terminal, the system transitions into the target tracking phase. During this phase, the FPGA control terminal dynamically adjusts commands to ensure that the echo frequency remains within the operational range of the photodetector, to compensate for the Doppler frequency drift caused by target motion while performing data acquisition and computation of the echo signal. By utilizing the short control cycle of the FPGA, the velocity of the tracked object is approximated as a constant value in a short period of time, enabling incremental adjustments of frequency offset.

**Figure 20:**
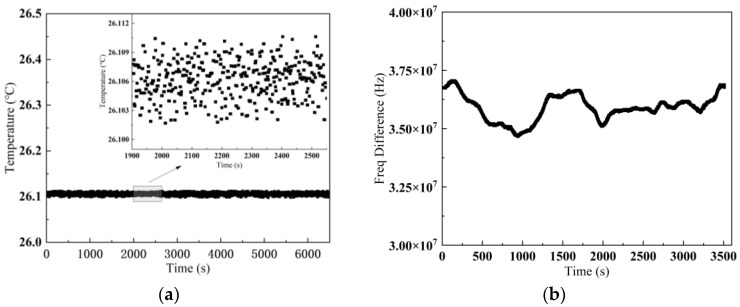
(**a**) Stabilized RL temperature variation; (**b**) stabilized frequency difference variation.

This experiment focuses on simulating frequency stability under conditions of constant frequency difference between the two lasers. The laser driver transmits real-time temperature data to the control terminal through serial interface, with its intervals set to 1 s while the post-processing frequency information is also displayed and recorded through serial monitoring software. The system stability testing yields comprehensive datasheets comprising over 6000 temperature data points and approximately 3500 frequency difference data points. Figure 20 illustrates the laser temperature and frequency offset fluctuations over a period, with an RMSE of 0.003 °C for temperature variations. The experimental data reveal that the temperature fluctuation is 0.007 °C, while the dual-laser frequency difference fluctuation is measured to be less than 3 MHz. These results validate the system’s capability to maintain precise frequency control during tracking, critical for a sustained target lock and accurate velocity and distance measurement.

### 4.4. Performance Validation of Moving Target Simulation

**Figure 21:**
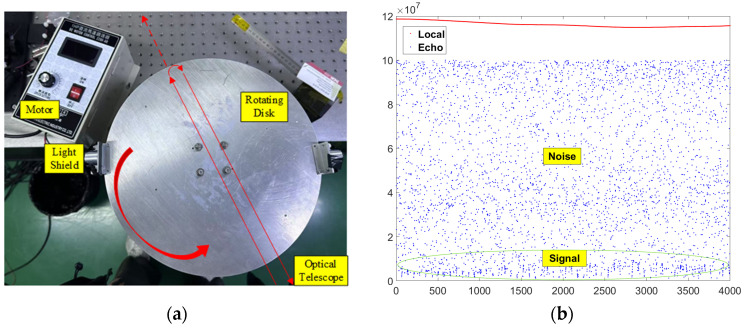
(**a**) Laboratory experiment platform; (**b**) local and echo frequency over time.

To evaluate the system performance, a rotating disk apparatus is employed under a laboratory environment to simulate a dynamic target scenario as shown in Figure 21. The experimental setup involved directing the output laser beam toward a metallic light shield mounted on the rotating disk, thereby creating intermittent target reflections analogous to moving objects. The disk is driven by a motor operating at 1800 RPM, producing echo signals with approximately 100 pW power level. The dual-frequency architecture presents inherent technical challenges due to the wavelength-pump current dependency and exceptional thermal sensitivity of the laser. Without stringent temperature regulation, the detection reliability of the LiDAR system would be compromised by thermal-induced wavelength drift. The experimental results reveal discernible velocity tracking profiles despite substantial noise components, which are attributed to the limited target size of the metallic light shield causing sporadic laser–target interactions.

The dual-loop cascade control system demonstrates robust performance by maintaining the echo signal within the 150 MHz operational bandwidth of the photodetector, ensuring uninterrupted target acquisition capabilities. The system achieves thermal equilibrium at the setpoint temperature within 3 s, which—coupled with the specified bandwidth of the detector—permits a theoretical maximum Doppler shift tolerance of 25 MHz/s, corresponding to an acceleration threshold of 26.6 m/s^2^.

However, operational constraints would arise considering real-world aerodynamic environments. Aircraft undergo aggressive maneuvering profiles and experience significant altitude variations, which induce pressure and temperature fluctuations despite the integrated voltage regulation and temperature stabilization subsystems within the LiDAR system. These environmental perturbations induce frequency excursions in the laser emission spectrum, thereby increasing the complexity of the cascade PID control loop. Consequently, the effective operational Doppler shift tolerance is anticipated to be reduced below the calculated theoretical limit under the actual airborne scenario.

### 4.5. Field Ranging of Stationary Target

**Figure 22:**
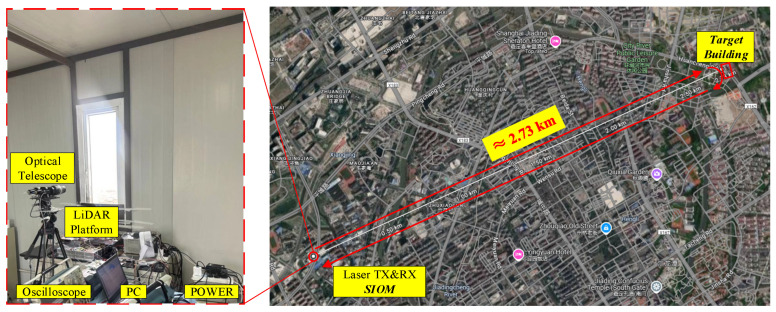
LiDAR ranging experiment between Shanghai Institute of Optics and Fine Mechanics (SIOM) and the target building.

Given the current experimental limitations, this study prioritized static-ranging experiments to evaluate the LiDAR system performance as illustrated in Figure 22. The experiment was conducted on a sunny afternoon with good visibility, under ambient conditions featuring atmospheric pressure of 1009 hPa, relative humidity of 42% and good air quality. The outdoor ambient temperature is approximately 32 °C, while the LiDAR system was maintained at a controlled room temperature of 25 °C. The target selected was a stationary building facade positioned 2.73 km away (exhibiting negligible Doppler shift). Employing the LiDAR system, target distance measurements were acquired over a 0.5 s data processing interval. The demonstrated novel integrated a dual-laser control system incorporating a laser driver, FPGA control module and data acquisition/processing terminal. This system ensured that echo signals remained within the detector bandwidth, facilitating reliable and consistent target information retrieval.

**Figure 23:**
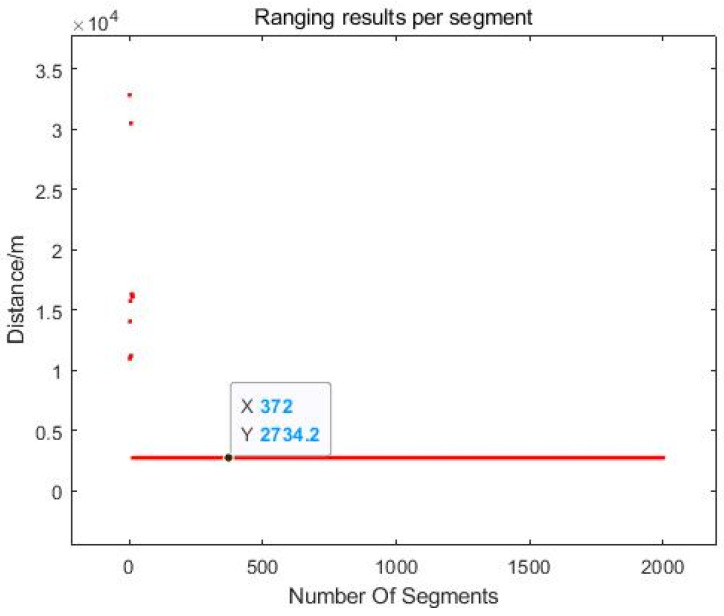
LiDAR experimental ranging results.

The integrated control system successfully constrained the echo frequency within the operational bandwidth of the balanced photodetector, achieving stable and accurate distance measurement. The experimental results shown in Figure 23 indicate the distance between the target building and the laboratory is 2.73 km, which closely matches the true value, demonstrating the prominent accuracy of the LiDAR system under static laboratory conditions. These findings underscore the enormous potential and robust performance of the system in real-world applications requiring precise remote detection.

## 5. Conclusions

This paper introduces an innovative compact laser driver architecture tailored for dual-frequency airborne coherent LiDAR systems, comprehensively elaborating on the system principles, laser characteristics, hardware implementation, and control algorithms. Unlike the commonly used low-voltage driver IC solutions, our discrete H-bridge and its peripheral circuit design efficiently drive high-power TECs integrated within the laser modules. Moreover, a novel cascade PID-control framework is employed to achieve precise control of the frequency difference between dual lasers, which not only greatly simplifies the complexity of hardware and software but also enhances the control robustness of the entire system. The flexible setting of host configurable parameters was achieved by customizing the data and instruction frame format between the control terminal and the laser driver, significantly improving the operational flexibility of the LiDAR system in varied operating environments. Experimental validation shows brilliant performance of the laser driver that successfully maintains temperatures within a tight 24.5–27.5 °C range, with a fluctuation below 0.007 °C, corresponding to dual-laser frequency difference fluctuations below 3 MHz. In addition, the linear constant current source demonstrates <1% long-term optical power stability, which fully satisfies the strict requirements for the demand of coherent LiDAR velocity and distance measurements.

Table 4 demonstrates a comparison and summary of the main characteristics of the laser drivers cited in the paper, showcasing the exceptional performance of the demonstrated laser driver in terms of temperature control and optical power stability.

Beyond meeting current demands for high-precision LiDAR, this work also provides a reference and establishes a foundational framework for the development of the next-generation integrated driver ICs. The novel architecture applied in this paper demonstrates high adaptability and robustness, which are key enabling factors for emerging applications in aerospace, autonomous navigation, and remote sensing. Specifically, its compact size combined with adaptive control capabilities meets the strict requirements of space-limited systems of the airborne LiDAR. By bridging the gap between high-performance laser control and practical system deployment, this study demonstrates the transformative potential of coherent LiDAR technologies in redefining precise measurement paradigms in various scientific and engineering fields. We believe that the high adaptability demonstrated in this paper ensures seamless integration with complex autonomous platforms, while the robust architecture ensures reliable operation in any environment. The proposed research results have significant prospects for promoting autonomous systems and space applications.

## Figures and Tables

**Table 1:** Electrical parameters of the integrated TEC.

Param	Value	Conditions (Vacuum)
$I_{max}$	1.8 A	$Qc=0,\;DT={DT}_{max},\;Th=50\;{}^{\circ}C$
$V_{max}$	9.6 V	$Qc=0,I=I_{max},Th=50\;{}^{\circ}C$
${{DT}_{max}}^{\;1}$	80 °C	$Qc=0,I=I_{max},Th=50\;{}^{\circ}C$
${{Qc}_{max}}^{\;2}$	0.4 W	$DT=0,I=I_{max},Th=50\;{}^{\circ}C$
${{Th}_{max}}^{\;3}$	200 °C	Maximum Processing Temperature

^1^ *DT*: Temperature Difference ^2^ *Qc*: Cooling Capacity ^3^ *Th*: Temperature of the Heat-sink Side of TEC.

**Table 2:** Electrical parameters of the TEC driver circuit.

Param	Value
TEC Driver Control Signal	0 V to 5 V
TEC Current Range ($-I_{max}$ to $I_{max}$)	−1.8 A to 1.8 A
TEC Voltage Range ($-V_{max}$ to $V_{max}$)	−7.2 V to 7.2 V
TEC Supply Voltage (VDD)	8 V
TEC Driver Control Signal	0 V to 5 V

**Table 3:** Electrical parameters of the constant current source.

Param	Value
Current Source Control Signal	0 V to 5 V
Supply Current	0 A to 3.6 A
Supply Voltage (VDD)	2.1 V

**Table 4:** A comparison and summary of the main characteristics of the laser drivers cited in the paper.

Authors	Temperature Fluctuations	Optical Power Fluctuations	Features
Li et al. [20]	<0.006 °C	Not reported	Hall–Libbrecht design-based
He et al. [21]	Not reported	<1%	Controllable closed-loop constant current feedback drive circuit, Neural PI control model
Zhao et al. [22]	<0.009 °C	Not reported	Mathematical model combining M sequence and differential evolution (DE) algorithms, Fuzzy PID algorithm
Gao et al. [23]	<0.01 °C	Not reported	PID algorithm
Yu et al. [24]	±0.005 °C	Not reported	FPGA based, high speed MOSFETs applied
Demonstrated	<0.007 °C Dual	<1%	Cascade PID algorithm, tunable parameters, FPGA-MCU integrated

## Data Availability

The raw data supporting the conclusions of this article will be made available by the authors on request.
